# The Danger of Walking with Socks: Evidence from Kinematic Analysis in People with Progressive Multiple Sclerosis

**DOI:** 10.3390/s20216160

**Published:** 2020-10-29

**Authors:** Su-Chun Huang, Gloria Dalla Costa, Marco Pisa, Lorenzo Gregoris, Giulia Leccabue, Martina Congiu, Giancarlo Comi, Letizia Leocani

**Affiliations:** 1 Neurorehabilitation Department and Experimental Neurophysiology Unit, INSPE-Institute of Experimental Neurology, San Raffaele Hospital, 20132 Milan, Italy; huang.suchun@hsr.it (S.-C.H.); dallacosta.gloria@hsr.it (G.D.C.); pisa.marco@hsr.it (M.P.); congiu.martina@hsr.it (M.C.); comi.giancarlo@hsr.it (G.C.); 2 Vita-Salute San Raffaele University, Via Olgettina, 58, 20132 Milan, Italy; gregorislorenzo96@gmail.com (L.G.); giulialeccabue95@gmail.com (G.L.)

**Keywords:** multiple sclerosis, gait analysis, kinematics, surface EMG, accelerator, inertial sensor, T10MW

## Abstract

Multiple sclerosis (MS) is characterized by gait impairments and severely impacts the quality of life. Technological advances in biomechanics offer objective assessments of gait disabilities in clinical settings. Here we employed wearable sensors to measure electromyography (EMG) and body acceleration during walking and to quantify the altered gait pattern between people with progressive MS (PwPMS) and healthy controls (HCs). Forty consecutive patients attending our department as in-patients were examined together with fifteen healthy controls. All subjects performed the timed 10 min walking test (T10MW) using a wearable accelerator and 8 electrodes attached to bilateral thighs and legs so that body acceleration and EMG activity were recorded. The T10MWs were recorded under three conditions: standard (wearing shoes), reduced grip (wearing socks) and increased cognitive load (backward-counting dual-task). PwPMS showed worse kinematics of gait and increased muscle coactivation than controls at both the thigh and leg levels. Both reduced grip and increased cognitive load caused a reduction in the cadence and velocity of the T10MW, which were correlated with one another. A higher coactivation index at the thigh level of the more affected side was positively correlated with the time of the T10MW (r = 0.5, *p* < 0.01), Expanded Disability Status Scale (EDSS) (r = 0.4, *p* < 0.05), and negatively correlated with the cadence (r = −0.6, *p* < 0.001). Our results suggest that excessive coactivation at the thigh level is the major determinant of the gait performance as the disease progresses. Moreover, demanding walking conditions do not influence gait in controls but deteriorate walking performances in PwPMS, thus those conditions should be prevented during hospital examinations as well as in homecare environments.

## 1. Introduction

Multiple sclerosis (MS) is a complex autoimmune disease characterized by multifocal and recurrent formation of demyelinating plaques possibly involving every area of the central nervous system (CNS) [1]. The disease usually affects multiple domains, but the motor pathway is constantly involved and usually it follows a disto-proximal gradient of severity with lower limbs being more precariously and severely impaired than the upper limbs [2]. Gait is one of the most disabling neurological symptoms in people with MS [3]. In the most advanced phases of the disease, gait dysfunction is typically due to pyramidal deficits, and sensory and cerebellar disturbances also coexist with variable extents [4].

Technological advances in biomechanics offer possibilities to objectively estimate gait disabilities such as joint kinematics, kinetics, and patterns of muscle activations during walking [5]. Non-invasive wireless wearable devices for the recording of surface electromyography and kinematics are commercially available [6]. These devices can be placed on different parts of the body based on clinical needs and they are capable of measuring gait quality in an everyday environment. For example, belt-mounted devices are used routinely in the clinical setting as they provide information on gait parameters such as cadence and velocity. The results of these simple techniques are highly consistent with more sophisticated laboratory equipment used in the research setting [7]. However, for people with MS the ideal conditions under which gait should be tested need to be better explored. Although for one of the most widely used walking tests, the 25 foot walk test, it is recommended to use comfortable shoes, in the clinical setting it can be useful to observe gait patterns without shoes in order to better appreciate subtle abnormalities in ankle or toe movements, and in order to reduce variability at longitudinal assessments due to changes of shoes. Even in research settings, gait parameters are collected during barefoot walking [8]. Sometimes, the barefoot condition is not fully reached and the patient is allowed to wear socks for better comfort. However, walking while wearing socks may be dangerous. Walking in socks without shoes or in slippers without a sole has been associated with falls in women [9]. Being barefoot or wearing socks without shoes may also increase the risk of falls from slipping or trauma from unexpected contact [10]. Older people going barefoot, wearing socks without shoes, or wearing slippers have an increased risk of serious injury at home due to a fall [11]. Falls are a common cause of harm in people with MS; it has been described that 50% of patients report falls in a 3-month period [12]. Walking under more demanding conditions, such as during cognitive loads, greatly influences motor performance [13]. Worsening of gait during a dual-task situation is also associated with an increased risk of falls in people with MS [8]. The aim of the current study was to explore whether walking with socks may be associated with a worsening in gait performance in people with progressive MS (PwPMS), similarly to what was already described for cognitive load.

## 2. Materials and Methods

### 2.1. Subjects

We examined gait performance in 40 consecutive patients attending the Department of Neurorehabilitation of San Raffaele Hospital (Milan, Italy), with a confirmed diagnosis of MS based on the 2017 McDonald criteria [14], age 18–65 years old, Expanded Disability Status Scale (EDSS) up to 6.5 (able to walk for at least 10 min safely with or without aids), absence of orthopedic pathologies that might influence walking performances, without depression nor cognitive involvements as per routine neurological and cognitive examinations at entry (token test, symbol digit modalities test, Beck Depression Inventory-II). All patients’ data were collected as part of their clinical care according to the Guideline of Good Clinical Practice [15]; all patients provided written informed consent to the use of their data for research. Fifteen healthy subjects were enrolled as the control group with similar age and sex distribution; they first provided written informed consent to participate in the study, that was approved by our Institutional Ethics Committee (approval number: N13/2017) and all data were anonymized prior to analysis.

### 2.2. Gait Analysis

Gait analysis was assessed using a G-Walk (BTS bioengineering, Italy), an inertial sensor which measures tri-axial accelerations, while performing a timed 10 min walking test (T10MW). The subject was asked to walk straight for 10 min with the sensor attached to the waist with a belt, covering the lower lumbar area (L4–L5). Body accelerations along the anterior-posterior, medio-lateral, and vertical axes during the T10MW were recorded with a sampling frequency of 100 Hz. According to standard T10MW procedure, patients were asked to complete the test at the maximum speed they could safely walk. A counting dual-task (DT) condition was also tested by adding a mental tracking cognitive task in which the subject was asked to count backward by 3 from 100 while performing the T10MW. As the dual task is routinely performed to test interference on gait by cognitive load, it was performed only in the more frequently used condition, while wearing shoes. Therefore, the T10MW was assessed in three different conditions: (1) walking with shoes on (2) walking with only socks on and (3) walking with shoes on while performing DT.

The acceleration data were analyzed with G-Studio (G-Studio software, BTS bioengineering, Milan, Italy) for the three conditions. Time, cadence, velocity, and step length were calculated for further analyses.

### 2.3. sEMG Recording

Surface EMG was used to record muscular activity simultaneously with the acceleration measurement. Eight wireless electrodes (FREEEMG-1000, BTS bioengineering, Milan, Italy) were attached directly to the skin overlying the Rectus Femoris (RF), long head of Biceps Femoris (BF), Tibialis Anterior (TA) and Medial Gastrocnemius (GM) bilaterally. EMG data were sampled at a rate of 1000 Hz and signals were remotely transferred to a USB receiver.

The sEMG data were processed with self-developed Matlab scripts (Matlab 2016b, MathWorks, Natickm, MA). The raw sEMG data were first band-pass filtered between 10 to 500 Hz, full-wave rectified, then smoothed with a low pass filter (3.5 Hz cut-off frequency). The averaged amplitude of the resting EMG (before the subject started walking) was subtracted from the smoothed EMG for each muscle. This subtracted data were then normalized to the largest recorded value of each muscle (max. EMG). Coactivation between each agonistic-antagonistic muscle pairs (RF-BF and TA-GM) were quantified as the coactivation index (CoI). The CoI was calculated as the overlap areas of the normalized EMG data divided by the duration of the overlapping, a higher CoI indicates more coactivation between the muscle pairs [16]. However, the length of the EMG data differs between subjects since it depends on the walking speed. In order to obtain a more stable CoI and enable the comparisons between subjects, CoI was calculated from five consecutive steps of the T10MW.

### 2.4. Clinical Assessment

The EDSS score was evaluated by the treating neurologist at hospital admissions. All patients also underwent a clinical evaluation of spasticity according to the Modified Ashworth Scale (MAS) on bilateral RF, BF, TA and GM. On the same muscular groups, strength was measured with Medical Research Council Scale (MRC) [17,18]. These scores were used to define the less affected (LA) and more affected (MA) side in each patient. Static balance performance was assessed with the Berg Balance Scale (BBS) [19].

Patient reported outcomes were also added to the clinical evaluation. The walking status of PwPMS was measured with a 12-Item MS Walking Scale (MSWS-12) [20]; fatigue was assessed with the Fatigue Severity Scale (FSS) [21]; the MS Spasticity Scale-88 (MSSS-88) and the Numeric Rating Scale of Spasticity (NRS) were used for estimating the impact of spasticity [22] on physical performances [23]. The risk of falls was evaluated with Conley scale [24]. The disability is evaluated using the Functional Independence Measure (FIM) [25] and the Barthel Index [26].

### 2.5. Statistics

For data demographics, the data are expressed in mean and standard deviations (SD). Independent t-test and chi-square tests were used to compare age, gender, and body mass index (BMI) distributions between PwPMS and healthy controls (HCs) respectively.

The spatiotemporal parameters and CoI from both PwPMS and HCs for all three conditions (shoes, socks, DT) were used for further statistics. Mixed two-way ANOVA (group x conditions) were employed to test significant differences between PwPMS and HCs under the three conditions. The analyses of CoI were performed in less and more affected side (LA/MA), respectively. If the ANOVA model was significant, turkey post-hoc analysis with Bonferroni correction (*p* < 0.01) was used to search the difference between (1) shoes and socks condition and (2) shoes and the DT condition, while the difference between PwPMS and HCs were tested with the independent t-test.

Correlations were performed among the kinematic parameters, sEMG recording, and clinical assessments. The alpha-level was corrected with Bonferroni correction and set at 0.017 for two tails as the correlations were performed in three major categories. Spearman’s correlation was used to explore the relationship between the quantitative data (spatiotemporal parameters and CoI) and the clinical assessment (EDSS, MSWS-12, FSS, MSSS-88, Conley, Barthel, FIM, BBS, and NRS). Pearson’s correlation was used to examine the relationship between spatiotemporal parameters and CoI. All the statistical analyses were performed with Prism 5 (GraphPad Software, Inc., San Diego, CA).

## 3. Results

### 3.1. Subjects Demographics

Forty people with progressive MS (20 males; mean age: 51.0 ± 9.8 years; mean BMI = 24.0 ± 4.6) with a mean EDSS score of 5.5 ± 1 (ranging from 1.5 to 6.5) were examined and fifteen HCs (4 males; mean age: 52.7 ± 4.4 years; mean BMI = 24.0 ± 2.2) were enrolled. The characteristics and results of clinical assessments of the two groups are shown in Table 1. No significant difference was found in age (*p* = 0.4971), sex (*p* = 0.3381), nor BMI (*p* = 0.9448) distributions between the groups.

### 3.2. Comparisons of Spatiotemporal Parameters

Significant differences in both groups (PwPMS and HCs) and conditions (shoes, socks, and DT) were found in time (group: *p* < 0.0001; condition: *p* = 0.0032), cadence (group: *p* < 0.0001; condition: *p* = 0.0032), velocity (both *p* < 0.0001) and step length (both *p* < 0.0001). Interactions between group and conditions were only significant in time (*p* = 0.0277) and step length (*p* = 0.0105). Post-hoc analyses revealed that compared with the HCs, the PwPMS showed longer time, lower cadence, slower velocity, and shorter step length while performing T10MW in all three conditions (*p* < 0.01 for all post-hoc comparisons).

For intra-group comparisons, when wearing shoes, PwPMS showed shorter time (*p* < 0.0001), higher cadence (*p* = 0.0005), higher velocity (*p* < 0.0001), and longer step length (*p* < 0.0001) than walking with socks. On the other hand, HCs showed only smaller step size when wearing socks compared to shoes (*p* = 0.0142).

For the comparison between single and dual tasks, longer time (*p* < 0.0001), lower velocity (*p* = 0.0002), and shorter step length (*p* = 0.0003) was found in PwPMS while performing a counting DT, while cadence was not significantly different (*p* = 0.09). Interestingly, significantly reduced cadence during the DT compared to a single task was found in HCs (*p* = 0.014).

**Figure 1:**
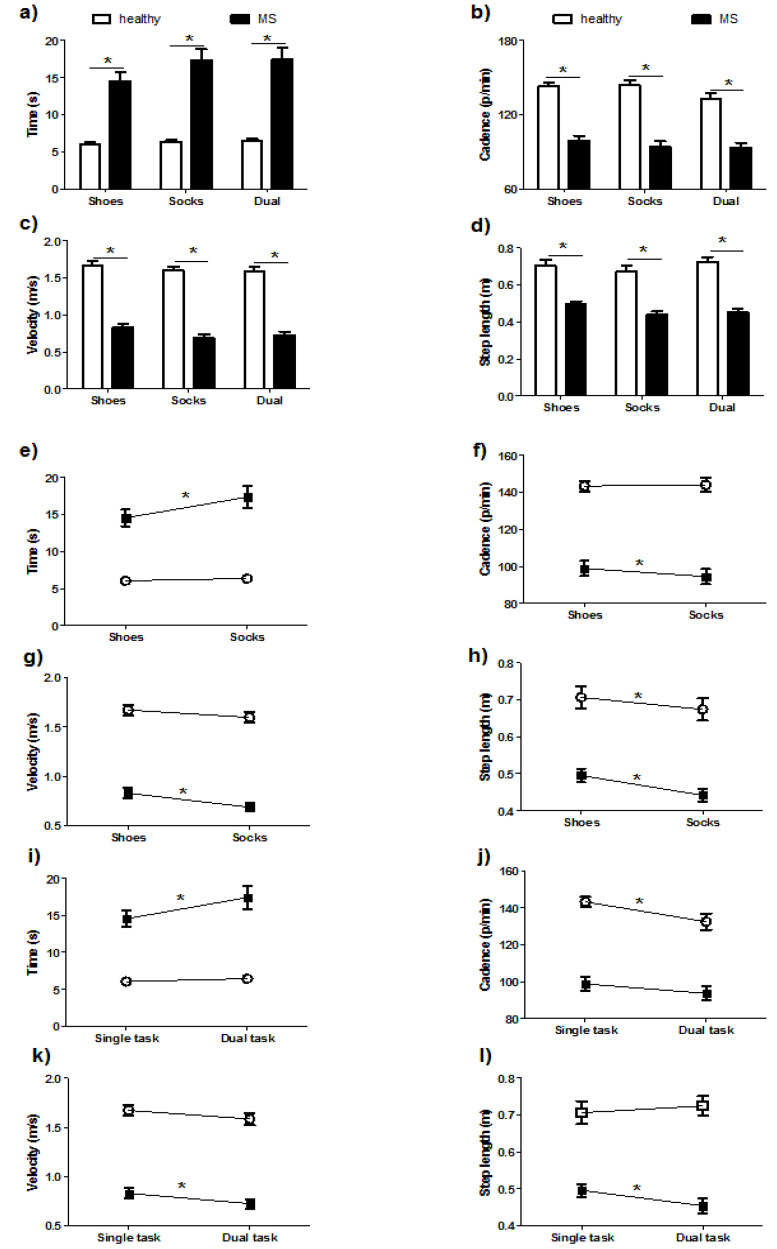
Inter- and intra group comparisons of spatiotemporal parameters. Significant group differences in time (**a**), cadence (**b**), velocity (**c**) and step size (**d**) were found in all three conditions. Intra-group comparison between shoes and socks conditions also showed significant differences in time (**e**), cadence (**f**), velocity (**g**), and step length (**h**) in PwPMS, while only step length in the HCs. For the comparison of single and dual tasks, longer time (**i**), lower velocity (**k**), and shorter step length (**l**) was found when PwPMS were performing a DT compared to performing a single task, while in the HCs only significantly reduced cadence (**j**) was found. *: *p* < 0.01 in post hoc analysis ((**a**–**d**): between-group comparison; (**j**–**l**): within-group comparisons).

The results of spatiotemporal parameters are shown in Figure 1.

### 3.3. Comparisons of Coactivation Index

Two-way ANOVA showed significant group differences of coactivation in both the MA and LA side in RF-BF (*p* = 0.0007 for both) and GM-TA (MA: *p* = 0.0144; LA: *p* = 0.0047) pairs, while no significant difference were found among conditions. Post-hoc analyses revealed that compared with HCs, PwPMS showed higher coactivation in the MA and LA sides for both antagonistic pairs among all three conditions (*p* < 0.01 for all).

**Figure 2:**
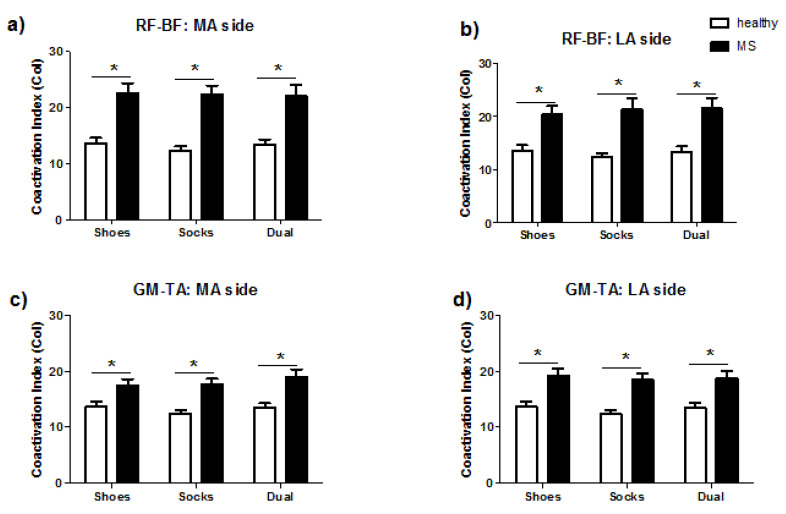
The inter-group difference of the coactivation index. Two-way ANOVA showed significant group difference of coactivation in both the MA and LA side in RF-BF (**a**,**b**) and GM-TA (**c**,**d**), while no significant difference was found among conditions. *: *p* < 0.01 in posthoc analyses.

For intra-group comparisons, in both PwPMS and the HC, no difference of the CoI was found when comparing between shoes and socks conditions, nor in shoes and with DT conditions. The results are shown in Figure 2.

### 3.4. Correlations among Measurements

For correlation between clinical assessments and spatiotemporal parameters, the EDSS score was correlated with time (shoes: r = 0.4785, *p* = 0.0018; socks: r = 0.4984, *p* = 0.0011; DT: r = 0.4006, *p* = 0.0104), cadence (shoes: r = −0.4932, *p* = 0.0012; socks: r = −0.4995, *p* = 0.0010; DT: r = −0.4270, *p* = 0.0060), and velocity (shoes: r = −0.4790, *p* = 0.0018; socks: r = −0.4967, *p* = 0.0011; DT: r = −0.4225, *p* = 0.0066) in all three conditions. On the other hand, with EMG results, the EDSS correlated with the CoI in the RF-BF pair of the MA side in socks (r = 0.4237, *p* = 0.0169) and with DT (r = 0.4761, *p* = 0.0078) conditions, also a trend correlation was found in the shoes condition (r = 0.3828, *p* = 0.0368). The FSS was correlated with the CoI in the RF-BF pair in both the MA and LA side in the socks condition (MA: r = −0.4953, *p* = 0.0054; LA: r = −0.5457, *p* = 0.0105). The FIM scores was correlated with time in shoes (r = −0.4026, *p* = 0.0100) and with the DT (r = −0.3875, *p* = 0.0135), and with velocity in all three conditions (shoes: r = 0.4080, *p* = 0.0090; socks: r = 0.4011, *p* = 0.0103; DT: r = 0.4235, *p* = 0.0065). The BBS correlated with time (r = −0.3961, *p* = 0.0126) and velocity (r = 0.3957, *p* = 0.0127) in shoes condition. The correlation results are summarized in Table 2 and Table 3.

**Figure 3:**
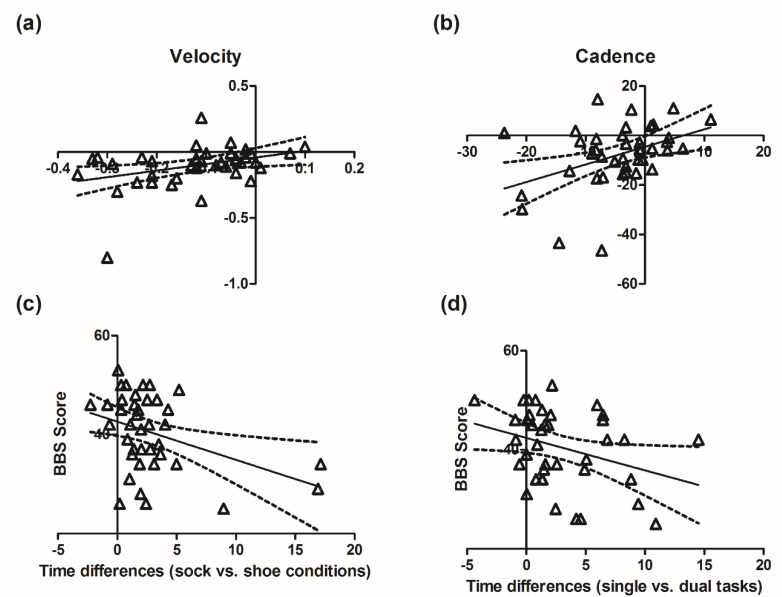
The correlations among kinematics and clinical assessments when the walking conditions changed for PwPMS. (**a**,**b**): x-axis: the difference between the shoes and the socks conditions (socks minus shoes); y-axis: the difference between the single task and DT conditions (single minus DT, both performed with shoes). Positive correlations were found in velocity (r = 0.3662, *p* = 0.0141) and cadence (r = 0.4158, *p* = 0.0076) between the differences of the shoes vs. socks condition and the single vs. DT conditions. (**c**,**d**): A significant negative correlation was found between the BBS scores and the time increases from shoe to sock conditions (r = −0.3901, *p* = 0.0141), and a trend correlation was found with time increases of single versus dual tasks (r = −0.3247, *p* = 0.0437).

Finally, when we compared the changes from the standard shoe condition to the two challenging conditions (i.e., socks or DT), we found significant positive correlations between the reduction in velocity (r = 0.3662, *p* = 0.0141) and cadence (r = 0.4158, *p* = 0.0076) when walking with socks versus shoes and when performing the dual versus simple task for PwPMS. A negative correlation was found between lower score of the BBS and the time increases from shoe condition to the socks condition (r = −0.3901, *p* = 0.0141) and a trend of negative correlation with the time increases from a single to a DT condition (r = −0.3247, *p* = 0.0437). The results are shown in Figure 3. No such correlations were found in HCs.

## 4. Discussion

In the present study we employed wireless wearable devices to examine changes in gait control in demanding conditions. The T10MW was tested under three conditions (shoes, socks, and the counting DT) with the combined use of an accelerator and sEMG monitoring. As expected, PwPMS showed worse gait performance in kinematics and higher coactivation in antagonistic muscle pairs at thigh and leg levels than HCs. We found that only in the PwPMS, the kinematic measures changed when walking was performed under demanding conditions, while the pattern of coactivation remained the same. Further, the kinematic changes between the socks versus shoes conditions were positively correlated with those found between the single versus dual task condition. This result indicates that for PwPMS, the impact on gait performance when walking with socks without shoes, is correlated with that introduced by a cognitive load; similar findings were not present in the HC group. Both walking with socks and with a cognitive load were associated with increased risk of falls in women, elder people, or in people with MS [8,9,10,11].

A score of less than 45 in the Berg Balance Scale exposes one to a greater risk of falling [27]. We found that in the PwPMS group, lower scores of the Berg Balance Scale were related to worse gait control when walking in socks or performing a counting DT. No difference nor correlation was found regarding gender, age, nor BMI in different conditions. Therefore, for tests such as the T10MW or the timed 25 foot walk test, performing with socks should be avoided for all PwPMS.

The sEMG results showed increased coactivation in both the MA and LA side of the lower limbs during total stance in PwPMS compared with the HCs. However, due to the limitation of the devices used, the stance cannot be further separated into sub-phases. Boudarham et al. reported higher coactivation during the whole stance at the leg level, while at the thigh level only during the single support phase [28]. Compared with their group, our PwPMS group has a higher disability (mean EDSS: 5.5 vs. 3.8) and worse spasticity (MAS of MA side: 2.2 vs. 1), which could explain why we found excessive coactivation in the whole lower limbs. Also, in Budarham’s study, no correlation between the EDSS and the CoI was found, while in our study the CoI of RF-BF at the MA side was correlated with the EDSS in more challenging walking conditions (i.e., with socks or the counting DT). Furthermore, the CoI of RF-BF at both the MA and LA side were correlated with worsening of most kinematic measurements. These results suggest that as the disease progresses, higher coactivation at the thigh level is the major source of the increasing walking impairment.

It is important to combine the information regarding muscle activation and joint kinematics to have a more comprehensive view of gait performance, which is fundamental for the clinicians to design more tailored rehabilitation protocols [29]. Thanks to the advance of technology, both sEMG and kinematics can be measured with wearable devices in the clinical setting. Additionally, wireless communication allows the remote transfer of data to laboratories and clinics for further analysis. This approach paves the way for remote assessment, as it is able to provide real-time information for both the patient and the clinician [6]. The costs for wearable devices used routinely in the clinical setting are usually lower compared with more sophisticated non-wearable equipment reserved to research laboratory environment [6]. These potential advantages make wearable devices a good candidate to be incorporated into home care and remote medicine, besides the hospital settings.

There are some limitations of the current study. First, the inertial sensor is not sensitive enough to reliably distinguish sub-phases of the stance cycle. All the data were reported as the performance of a whole gait cycle. However, for patients with a milder disability, the differences may only appear during the sub-phases. Second, as our cohort of patients was already characterized by moderate EDSS severity already indicating involvement of gait, we could not test whether kinematic parameters in the present study may be more sensitive than a clinical examination. Third, as the dual task has been performed with shoes, it is not possible to explore possible further worsening of gait measures under the combination of the two more difficult conditions (i.e., dual task with socks). Last, as the majority of our cohort included subjects with normal BMI, our results may not fully reflect the whole BMI variability.

## 5. Conclusions

Walking tests wearing socks should be discouraged to prevent falls for PwPMS. This concern should be embedded into guidelines for future remote medicine when these measurements can be performed during home care instead of hospital settings. The combined use of wearable accelerators and sEMG provide quantitative measurements of muscle activity and kinematics during walking, which can benefit future remote medicine programs, offering the opportunity to monitor disease progression and evaluate the efficiency of rehabilitation for PwPMS remotely.

## Figures and Tables

**Table 1:** Data demographics of the subjects and clinical assessments. No significant difference was found in gender (*p* = 0.3381), age (*p* = 0.4971) nor in body mass index (BMI, *p* = 0.9448) between groups. Data are shown in mean ± standard deviation format. PwPMS: patients with progressive multiple sclerosis; HCs: healthy controls. MAS: Modified Ashworth Scale; MRC Scale: Medical Research Council Scale; MSWS-12: 12-Item MS Walking Scale; FSS: Fatigue Severity Scale; MSSS-88: MS Spasticity Scale-88; FIM: Functional Independence Measure; BBS: Berg Balance Scale; NRS: Numeric Rating Scale of Spasticity.

Characteristics	PwPMS (n = 40)	HC (n = 15)
Gender (M/F)	20 / 20	4 / 9
Age (years)	50.9 ± 9.8	52.7 ± 4.4
BMI	24.0 ± 4.6	24.0 ± 2.2
EDSS	5.5 ± 1.1	-
More Affected Side (R/L)	23 / 17	-
MAS (more affected side)	2.4 ± 2.0	-
MRC scale (more affected side)	13.1 ± 3.2	-
MSWS-12	38.6 ± 9.7	-
FSS	39.5 ± 15.0	-
MSSS-88	188.6 ± 52.7	-
Conley scale	2.9 ± 1.8	-
Barthel scale	88.4 ± 10.3	-
FIM	112.5 ± 9.0	-
BBS	40.5 ± 7.7	-
NRS	3.9 ± 2.6	-

**Table 2:** Spearman’s correlation coefficient between spatiotemporal parameters and clinical assessments in all the conditions. Spearman’s correlation was performed to explore the relationship between kinematics and clinical measurements.

Variables	Conditions	EDSS	MSWS-12	FSS	MSSS-88	Conley	Barthel	FIM	BBS	NRS
Time	shoes	0.48 **	0.22	−0.23	−0.01	−0.07	−0.11	−0.40 *	−0.40 *	0.05
(N = 40)	socks	0.50 **	0.22	−0.27	−0.04	−0.08	−0.01	−0.35	−0.32	0.06
	DT	0.40 *	0.15	−0.16	0.03	−0.16	−0.00	−0.39 *	−0.29	−0.03
Cadence	shoes	−0.49 **	−0.20	0.31	0.08	0.11	0.08	0.33	0.36	0.07
(N = 40)	socks	−0.50 **	−0.21	0.32	0.06	0.13	0.02	0.33	0.35	0.07
	DT	−0.43 **	−0.13	0.15	−0.09	0.14	−0.07	0.29	0.27	0.16
Velocity	shoes	−0.48 **	−0.22	0.23	0.02	0.06	0.10	0.41 **	0.40 *	−0.05
(N = 40)	socks	−0.50 **	−0.20	0.27	0.03	0.11	0.03	0.40 *	0.36	−0.07
	DT	−0.42 **	−0.13	0.18	0.01	0.11	0.06	0.42 **	0.34	−0.08
Step	shoes	−0.27	−0.03	0.24	0.13	0.08	0.06	0.32	0.24	−0.13
Length	socks	−0.18	−0.06	0.14	0.03	−0.01	−0.04	0.11	0.04	−0.21
(N = 40)	DT	−0.15	−0.08	0.11	0.02	0.12	0.03	0.30	0.14	−0.13

The significance level was set to *p* < 0.017 *: *p* < 0.017; **: *p* < 0.001.

**Table 3:** Spearman’s correlation coefficient between the coactivation index and clinical assessments in all the conditions. Spearman’s correlation was performed to explore the relationship between sEMG recording and clinical exams.

Variables	Conditions	EDSS	MSWS-12	FSS	MSSS-88	Conley	Barthel	FIM	BBS	NRS
RF-BF MA	shoes	0.38	0.06	−0.27	−0.03	−0.10	−0.13	−0.30	−0.15	−0.41
(N = 31)	socks	0.42 *	−0.10	−0.50 **	−0.11	−0.18	−0.02	−0.14	−0.12	−0.30
	DT	0.48 **	−0.04	−0.38	−0.13	−0.05	0.05	−0.08	0.02	−0.35
RF-BF LA	shoes	0.12	−0.28	−0.46	−0.21	−0.09	−0.02	−0.13	0.02	−0.22
(N = 22)	socks	0.21	0.07	−0.55 *	−0.18	0.03	−0.05	−0.12	0.02	−0.02
	DT	0.26	−0.32	−0.41	−0.13	−0.08	−0.01	−0.00	0	−0.05
GM-TA MA	shoes	0.13	−0.04	0.02	0.16	−0.06	0.20	−0.03	0.10	0.17
(N = 40)	socks	0.07	0.02	−0.01	0.07	0.06	0.14	−0.08	0.01	0.13
	DT	0.17	−0.02	0.01	0.02	−0.05	0.22	−0.07	0.01	0.12
GM-TA LA	shoes	0.22	0.07	−0.09	0.07	0.11	0.12	−0.34	−0.39	0.18
(N = 40)	socks	0.16	0.10	0.13	0.18	0.33	0	−0.28	−0.30	0.22
	DT	0.02	−0.10	−0.11	−0.01	0.22	0.03	−0.14	−0.18	0.27

The significance level was set to *p* < 0.017. *: *p* < 0.017; **: *p* < 0.001.

## References

[1] Lassmann H., Bruck W., Lucchinetti C.F. (2007). The immunopathology of multiple sclerosis: An overview. Brain Pathol..

[2] Joy J.E., Johnston R.B., Institute of Medicine (US) Committee on Multiple Sclerosis (2001). Current Status and Strategies for the Future. Multiple Sclerosis: Current Status and Strategies for the Future.

[3] Heesen C., Böhm J., Reich C., Kasper J., Goebel M., Gold S.M. (2008). Patient perception of bodily functions in multiple sclerosis: Gait and visual function are the most valuable. Mult. Scler. J..

[4] Kalron A., Givon U. (2016). Gait characteristics according to pyramidal, sensory and cerebellar EDSS subcategories in people with multiple sclerosis. J. Neurol..

[5] Lizama L.E.C., Khan F., Lee P.V., Galea M.P. (2016). The use of laboratory gait analysis for understanding gait deterioration in people with multiple sclerosis. Mult. Scler. J..

[6] Shanahan C.J., Boonstra F.M.C., Lizama L.E.C., Strik M., Moffat B.A., Khan F., Kilpatrick T., Van Der Walt A., Galea M.P., Kolbe S.C. (2018). Technologies for Advanced Gait and Balance Assessments in People with Multiple Sclerosis. Front. Neurol..

[7] Vítečková S., Horáková H., Poláková K., Krupička R., Růžička E., Brožová H. (2020). Agreement between the GAITRite® System and the Wearable Sensor BTS G-Walk® for measurement of gait parameters in healthy adults and Parkinson’s disease patients. PeerJ.

[8] Etemadi Y. (2016). Dual task cost of cognition is related to fall risk in patients with multiple sclerosis: A prospective study. Clin. Rehabil..

[9] Larsen E.R., Mosekilde L., Foldspang A. (2004). Correlates of falling during 24 h among elderly Danish community residents. Prev. Med..

[10] Menz H.B., Morris M.E., Lord S.R. (2006). Footwear Characteristics and Risk of Indoor and Outdoor Falls in Older People. Gerontology.

[11] Kelsey J.L., Procter-Gray E., Nguyen U.-S.D.T., Li W., Kiel D.P., Hannan M.T. (2010). Footwear and falls in the home among older individuals in the MOBILIZE Boston Study. Footwear Sci..

[12] Coote S., Sosnoff J.J., Gunn H. (2014). Fall Incidence as the Primary Outcome in Multiple Sclerosis Falls-Prevention Trials: Recommendation from the International MS Falls Prevention Research Network. Int. J. MS Care.

[13] Leone C., Patti F., Feys P. (2014). Measuring the cost of cognitive-motor dual tasking during walking in multiple sclerosis. Mult. Scler. J..

[14] Thompson A.J., Banwell B.L., Barkhof F., Carroll W.M., Coetzee T., Comi G., Correale J., Fazekas F., Filippi M., Freedman M.S. (2018). Diagnosis of multiple sclerosis: 2017 revisions of the McDonald criteria. Lancet Neurol..

[15] (2001). ICH Harmonised Tripartite Guideline: Guideline for Good Clinical Practice. J. Postgrad. Med..

[16] Unnithan V.B., Dowling J.J., Frost G., Ayub B.V., Bar-Or O. (1996). Cocontraction and phasic activity during GAIT in children with cerebral palsy. Electromyogr. Clin. Neurophysiol..

[17] Bohannon R.W., Smith M.B. (1987). Interrater Reliability of a Modified Ashworth Scale of Muscle Spasticity. Phys. Ther..

[18] Hermans G., Clerckx B., Vanhullebusch T., Segers J., Vanpee G., Robbeets C., Casaer M.P., Wouters P., Gosselink R., Berghe G.V.D. (2011). Interobserver agreement of medical research council sum-score and handgrip strength in the intensive care unit. Muscle Nerve.

[19] Toomey E., Coote S. (2013). Between-Rater Reliability of the 6-Minute Walk Test, Berg Balance Scale, and Handheld Dynamometry in People with Multiple Sclerosis. Int. J. MS Care.

[20] Hobart J.C., Riazi A., Lamping D.L., Fitzpatrick R., Thompson A.J. (2003). Measuring the impact of MS on walking ability: The 12-Item MS Walking Scale (MSWS-12). Neurology.

[21] Krupp L.B., LaRocca N.G., Muir-Nash J., Steinberg A.D. (1989). The fatigue severity scale. Application to patients with multiple sclerosis and systemic lupus erythematosus. Arch. Neurol..

[22] Hobart J.C., Riazi A., Thompson A.J., Styles I.M., Ingram W., Vickery P.J., Warner M., Fox P.J., Zajicek J. (2005). Getting the measure of spasticity in multiple sclerosis: The Multiple Sclerosis Spasticity Scale (MSSS-88). Brain.

[23] Farrar J.T., Troxel A.B., Stott C., Duncombe P., Jensen M.P. (2008). Validity, reliability, and clinical importance of change in a 0–10 numeric rating scale measure of spasticity: A post hoc analysis of a randomized, double-blind, placebo-controlled trial. Clin. Ther..

[24] Guzzo A., Meggiolaro A., Mannocci A., Tecca M., Salomone I., La Torre G. (2015). Conley Scale: Assessment of a fall risk prevention tool in a General Hospital. J. Prev. Med. Hyg..

[25] Ottenbacher K.J., Hsu Y., Granger C.V., Fiedler R.C. (1996). The reliability of the functional independence measure: A quantitative review. Arch. Phys. Med. Rehabil..

[26] Mahoney F.I., Barthel D.W. (1965). Functional Evaluation: The Barthel Index. Md. State Med. J..

[27] Berg K.O., Wood-Dauphinee S.L., Williams J.I., Maki B. (1992). Measuring balance in the elderly: Validation of an instrument. Can. J. Public Health.

[28] Boudarham J., Hameau S., Zory R., Hardy A., Bensmail D., Roche N. (2016). Coactivation of Lower Limb Muscles during Gait in Patients with Multiple Sclerosis. PLoS ONE.

[29] Papagiannis G.I., Triantafyllou A.I., Roumpelakis I.M., Zampeli F., Eleni P.G., Koulouvaris P., Papadopoulos E.C., Papagelopoulos P.J., Babis G.C. (2019). Methodology of surface electromyography in gait analysis: Review of the literature. J. Med. Eng. Technol..

